# Predictive value of 18F-FDG PET/CT delta radiomics model for prognosis after neoadjuvant therapy for locally advanced pancreatic cancer

**DOI:** 10.3389/fonc.2026.1902166

**Published:** 2026-07-30

**Authors:** Qiao-Liang Chen, Ling-Xiao Yang, Ai-Mei Li, Liang Mao, Yu-Dong Qiu, Juan Du, Jian He

**Affiliations:** 1 Department of Nuclear Medicine, Nanjing Drum Tower Hospital Clinical College of Nanjing University of Chinese Medicine, Nanjing, China; 2 Department of Hepatopancreatobiliary Surgery, Nanjing Drum Tower Hospital, Affiliated Hospital of Medical School, Nanjing University, Nanjing, China; 3 The Comprehensive Cancer Center, Nanjing Drum Tower Hospital, Affiliated Hospital of Medical School, Nanjing University, Nanjing, China

**Keywords:** delta radiomics, locally advanced pancreatic cancer, predictive model, prognosis, progression-free survival

## Abstract

**Purpose:**

To develop a delta radiomics model based on 18F-FDG PET/CT to predict prognosis after NAT for LAPC.

**Materials and methods:**

This study retrospectively collected data from 33 patients with LAPC admitted to our hospital from August 2019 to July 2024. The volume of the regions of interests (VOIs) of LAPC lesions was outlined on 18F-FDG PET/CT images before and after neoadjuvant therapy. Radiomics features were extracted based on the VOIs, and the subtraction of the two radiomics features was the delta radiomics feature. Feature screening was performed using t-test and LASSO-COX regression to build a prediction model. ROC curves were used to assess the predictive ability of the prediction model for progression-free survival (PFS). Calibration curve and decision curve analysis (DCA) were used to assess the accuracy and net benefit of the prediction model, respectively.

**Results:**

Univariate Cox regression analysis demonstrated that neither clinical parameters nor 18F-FDG PET/CT metabolic parameters were independent factors in predicting PFS in LAPC. A predictive model was constructed based on two delta radiomics features screened by LASSO-COX regression. The delta radiomics model demonstrated a high predictive ability for PFS with a C-index of 0.78 (95%CI: 0.68-0.88). High-risk patients identified by delta features showed markedly shorter PFS (HR:5.357, P = 0.006). DCA indicated favorable clinical utility for the delta radiomics model.

**Conclusion:**

The delta radiomics model based on 18F-FDG PET/CT, provides a non-invasive tool to predict prognosis in LAPC after NAT, which may facilitate personalized treatment strategies.

## Introduction

Pancreatic cancer (PC) is a clinically challenging malignancy with a generally poor prognosis and limited therapeutic efficacy, with locally advanced pancreatic cancer (LAPC) being particularly complex (1, 2). Because pancreatic cancer often remains asymptomatic at an early stage and is frequently diagnosed at an advanced or unresectable stage, early detection remains a major clinical challenge. Therefore, there is an urgent need for effective imaging-based biomarkers that may facilitate early diagnosis, treatment monitoring, and prognostic stratification (3, 4). LAPC is defined as pancreatic cancer in which the tumor has locally invaded or encircled major blood vessels but has not yet developed distant metastasis (5). In these patients, the difficulty of surgical resection is increased due to the large extent of tumor invasion, and the involvement of important blood vessels around the pancreas, such as the celiac artery and the superior mesenteric artery, is important to assess on imaging (5–7). Therefore, neoadjuvant therapy (NAT) is gradually being applied in the treatment of LAPC by administering chemotherapy or chemoradiotherapy before surgery, aiming to reduce the tumor size and increase the rate of surgical resection, thus improving the prognosis (1, 8). The prevailing treatment approach for patients with limited pancreatic cancer involves a combination of surgical resection and systemic chemotherapy (9, 10). In these patients, FOLFIRINOX or gemcitabine plus nab-paclitaxel chemotherapy is currently used as preoperative chemotherapy and is eventually combined with radiotherapy (11, 12).

18F-fluorodeoxyglucose (FDG) positron emission tomography/computed tomography (PET/CT) has been shown to acquire both functional and anatomical information in composite images.¹^8^F-FDG PET/CT has been widely applied in the evaluation of disease processes, including diagnosis, staging, monitoring of treatment efficacy, and detection of recurrence in many aspects (13–15).The value of 18F-FDG PET/CT technology lies not only in its ability to improve diagnostic accuracy, but also in its capacity to assess the biological characteristics of tumors and guide treatment decisions (16).

Conventional medical imaging is predominantly evaluated by physicians based on their experience and visual perception, both of which are subject to subjective interpretation. However, radiomics has been demonstrated to mitigate this influence through automation and quantitative analysis (17). The automated analysis of radiomics has been demonstrated to enhance the objectivity of diagnosis, while concomitantly improving the reproducibility and accuracy of diagnostic outcomes (18–20). The underlying principle of delta radiomics model entails the comparison of medical imaging data at disparate time points to identify features of imaging changes before and after treatment (21, 22). This approach relies on the capture of dynamic changes in the tumor to reveal biological responses and treatment effects (8, 23). Delta radiomics models have been demonstrated to exhibit superior capacity in capturing alterations within the tumor microenvironment consequent to therapeutic interventions, in comparison to conventional fixed time-point image analysis methodologies (24).

Therefore, the present study aimed to construct a delta radiomics model from pre- and post-NAT 18F-FDG PET/CT images of LAPC patients to investigate its predictive value for progression-free survival.

## Materials and methods

This study was approved by the institutional review board of our hospital. The requirement for informed consent was waived due to its retrospective design.

### Study population

Data were collected from 33 patients with LAPC who underwent to our hospital from August 2019 to July 2024. The standardized NAT protocol was administered to all patients, with a total of six cycles being completed. Both before and after NAT, the patients underwent 18F-FDG PET/CT examinations and eventually received surgical treatment. We followed up the patient continuously for 42 months. PFS was defined as the time from the initial NAT to the occurrence of disease progression in patients. Because this was a retrospective study, the Institutional Ethics Committee approved the study and waived the requirement of informed consent. Inclusion criteria: (1) The patients were diagnosed with pancreatic cancer following a pathology assessment and deemed to be LAPC by the attending clinician; (2) Prior to surgery, the patients underwent regular NAT; (3) The patients underwent 18F-FDG PET/CT scans before and after NAT, with the images obtained demonstrating adequate quality; (4) Age ≥ 18.Exclusion criteria: (1) The patients had received any form of antitumor therapy prior to the initial 18F-FDG PET/CT examination; (2) Tumors ware unable to be localized on 18F-FDG PET/CT images; (3) The patients had a combination of other malignancies; (4) Lost to follow-up.

### 18F-FDG PET/CT protocol and image assessment

All imaging procedures were performed using a Philips GXL16 integrated PET/CT system (Philips Healthcare, Netherlands). The radiochemical purity of 18F-FDG was determined to be greater than 95%. All patients fasted for at least 6 hours before administration, with capillary blood glucose levels rigorously monitored and maintained below 11.1 mmol/L. The 18F-FDG dosage was calculated based on body mass (5.18 MBq/kg) and administered via intravenous bolus injection. Following tracer injection, subjects remained in a supine resting position for 50–60 minutes to ensure proper biodistribution prior to image acquisition. Whole-body PET/CT scanning was conducted from the cranial vertex to the proximal femora using a sequential bed-position protocol with 2-minute acquisition per bed position. Concurrent CT imaging parameters included: 120 kVp tube voltage, 100 mA tube current, and 2.0 mm reconstructed slice thickness. CT-derived attenuation correction was applied to PET data, with subsequent image reconstruction performed using ordered subset expectation maximization (OSEM) algorithms (2 iterations, 8 subsets). All acquired datasets underwent quality assurance protocols to ensure diagnostic accuracy. Throughout the study period (August 2019 to July 2024), all PET/CT examinations were performed using the same Philips GXL16 PET/CT scanner. No scanner hardware upgrades, scanner replacement, or changes in image acquisition or reconstruction protocols occurred during the study period.

### Treatment protocol

All patients received at least two cycles of tislelizumab combined with the AG chemotherapy regimen, with each cycle lasting 21 days. On days 1 and 8 of each cycle, gemcitabine (1000 mg/m²) and nab-paclitaxel (125 mg/m²) were administered intravenously, while tislelizumab (200 mg) was given intravenously on day 1. Starting from the third cycle, stereotactic body radiation therapy (SBRT) was delivered, with a high-dose region receiving 50 Gy in 10 fractions and the remaining areas receiving 30 Gy in 10 fractions. Tumor response was assessed every two cycles according to RECIST 1.1 criteria. If the patient met surgical criteria, they proceeded to the surgical phase for radical pancreaticoduodenectomy.

### Image segmentation

All PET/CT images were initially exported in Digital Imaging and Communications in Medicine (DICOM) format. Before tumor segmentation, the original PET images were resampled to an isotropic voxel size of 4 × 4 × 4 mm and subsequently saved in compressed Neuroimaging Informatics Technology Initiative (.nii.gz) format. Gray-level discretization was performed using a fixed bin size (FBS) strategy with a bin width of 0.25. Identical preprocessing parameters were applied to all pre- and post-NAT PET images. Using the open-source software LIFEx (version 7.5.15, https://www.lifexsoft.org/), Physician A with 5 years of experience in nuclear medicine manually delineated the primary tumor volume of interests (VOIs) on the resampled PET images, and Physician B with 15 years of experience reviewed and corrected all segmentations by consensus. The VOIs were delineated using a fixed threshold of 40% of SUVmax.

### Radiomics feature extraction and selection

After images normalization, radiomics feature extraction was performed using the PyRadiomics package in Python 3.9. After feature extraction, features with zero variance were removed, and the remaining features were standardized using Z-score normalization. Delta radiomics feature was defined as the post-NAT radiomics feature value minus the pre-NAT radiomics feature value. Delta radiomics features were calculated as raw differences between post-NAT and pre-NAT radiomics feature values, defined as post-NAT feature value minus pre-NAT feature value. Relative percentage changes were not used because some radiomics features may have zero, negative, or near-zero values after normalization, which may lead to unstable ratio-based calculations. Therefore, absolute differences were adopted to provide a more stable and consistent representation of longitudinal imaging changes. Features significantly associated with PFS were screened using t-test (*P* < 0.05). A 10-fold cross-validation was then performed using the least absolute shrinkage and selection operator (LASSO)-COX, and the final feature set was automatically determined using the minimum λ as the best penalty in LASSO. The selected delta radiomics features were subsequently incorporated into logistic regression model.

### Statistical analysis

Univariate COX regression analysis was used to analyze the relationship between clinical parameters, 18F-FDG PET/CT metabolic parameters and PFS. The efficacy of the delta radiomics model in predicting PFS was evaluated using the time-dependent ROC curve. Calibration curves were used to evaluate accuracy, and decision curve analysis (DCA) was used to assess clinical net benefits. The model was subjected to high-low risk binary classification processing based on the optimal cut-off value, Kaplan–Meier curves and the log-rank test were used to compare PFS between the two groups. Cox proportional hazards regression was performed solely to estimate the hazard ratio and its 95% confidence interval for the high-risk group relative to the low-risk group.

## Results

### Patient characteristics and 18F-FDG PET/CT metabolic parameters

Based on the inclusion and exclusion criteria, a total of 33 patients (24 men and 9 women; median age: 60 years; range: 48–79 years; interquartile range: 56–66 years) were included in the retrospective study, with 17 patients (51.5%) in the progressive disease (PD) group and 16 patients (48.5%) in the non-progressive disease (non-PD) group. The clinicoradiological characteristics of all patients were summarized in **Table 1**. **Table 2** summarized the results of univariate and multivariate analyses. There were no significant differences in clinicoradiological variables between the PD group and non-PD group (all *P* > 0.05).

**Table 1:** Clinicoradiological characteristics of all patients, n (%).

Variables	Total (n = 33)
Age, median (IQR)	60.00 (56.00, 66.00)
Gender	
Female	9 (27.27)
Male	24 (72.73)
Long diameter before NAT, median (IQR)	3.30 (2.40, 4.00)
Short diameter before NAT, median (IQR)	2.10 (1.70, 2.80)
Long diameter after NAT, median (IQR)	1.90 (0.90, 3.00)
Short diameter after NAT, median (IQR)	1.30 (0.00, 1.80)
Delta long diameter, median (IQR)	0.42 (0.19, 0.70)
Delta short diameter, median (IQR)	0.40 (0.21, 1.00)
SUVmax, median (IQR)	6.94 (6.16, 9.02)
SUVmean, median (IQR)	4.11 (3.26, 5.02)
SUVpeak, median (IQR)	6.23 (5.21, 7.59)
SUVmin, median (IQR)	2.78 (2.46, 3.61)
MTV, median (IQR)	10.43 (7.30, 19.78)
TLG, median (IQR)	58.98 (32.27, 78.95)
ECOG	
0	19 (57.58)
1	14 (42.42)

NAT, neoadjuvant therapy; SUV, standardized uptake value; MTV, metabolic tumor volume; TLG, total lesion glycolysis; ECOG, Eastern Cooperative Oncology Group Performance Status.

**Table 2:** Univariate COX regression analysis of LAPC.

Variables	β	HR (95%CI)	P value
Age, median (IQR)	-0.01	0.99 (0.91 ~ 1.08)	0.782
Gender			
Female		1.00 (Reference)	-
Male	-0.71	0.49 (0.16 ~ 1.47)	0.205
Long diameter before NAT, median (IQR)	-0.03	0.97 (0.67 ~ 1.40)	0.867
Short diameter before NAT, median (IQR)	-0.18	0.84 (0.37 ~ 1.86)	0.661
Long diameter after NAT, median (IQR)	0.10	1.11 (0.77 ~ 1.58)	0.580
Short diameter after NAT, median (IQR)	0.23	1.26 (0.64 ~ 2.49)	0.504
Delta long diameter, median (IQR)	-0.33	0.72 (0.20 ~ 2.58)	0.612
Delta short diameter, median (IQR)	-0.28	0.75 (0.19 ~ 3.01)	0.689
SUVmax, median (IQR)	-0.07	0.93 (0.82 ~ 1.06)	0.296
SUVmean, median (IQR)	-0.11	0.89 (0.72 ~ 1.11)	0.311
SUVpeak, median (IQR)	-0.08	0.92 (0.79 ~ 1.07)	0.301
SUVmin, median (IQR)	-0.17	0.84 (0.61 ~ 1.16)	0.296
MTV, median (IQR)	0.00	1.00 (0.94 ~ 1.07)	0.947
TLG, median (IQR)	-0.01	0.99 (0.98 ~ 1.01)	0.358
ECOG			
0		1.00 (Reference)	-
1	0.78	2.17 (0.68 ~ 6.90)	0.189

NAT, neoadjuvant therapy; SUV, standardized uptake value; MTV, metabolic tumor volume; TLG, total lesion glycolysis; ECOG, Eastern Cooperative Oncology Group Performance Status.

### Radiomics model

Total of 1037 radiomics features were extracted. Following a thorough examination of the data through t-test and LASSO-COX regression analyses, the two delta radiomics features that demonstrated the greatest efficacy in predicting progression-free survival (PFS) were identified. These features, namely wavelet_LHH_firstorder_Kurtosis and wavelet_HHH_gldm_DependenceVariance, were then incorporated into a logistic regression model to construct the delta radiomics model.

### Model performance evaluation and application for risk stratification

**Figure 1:**
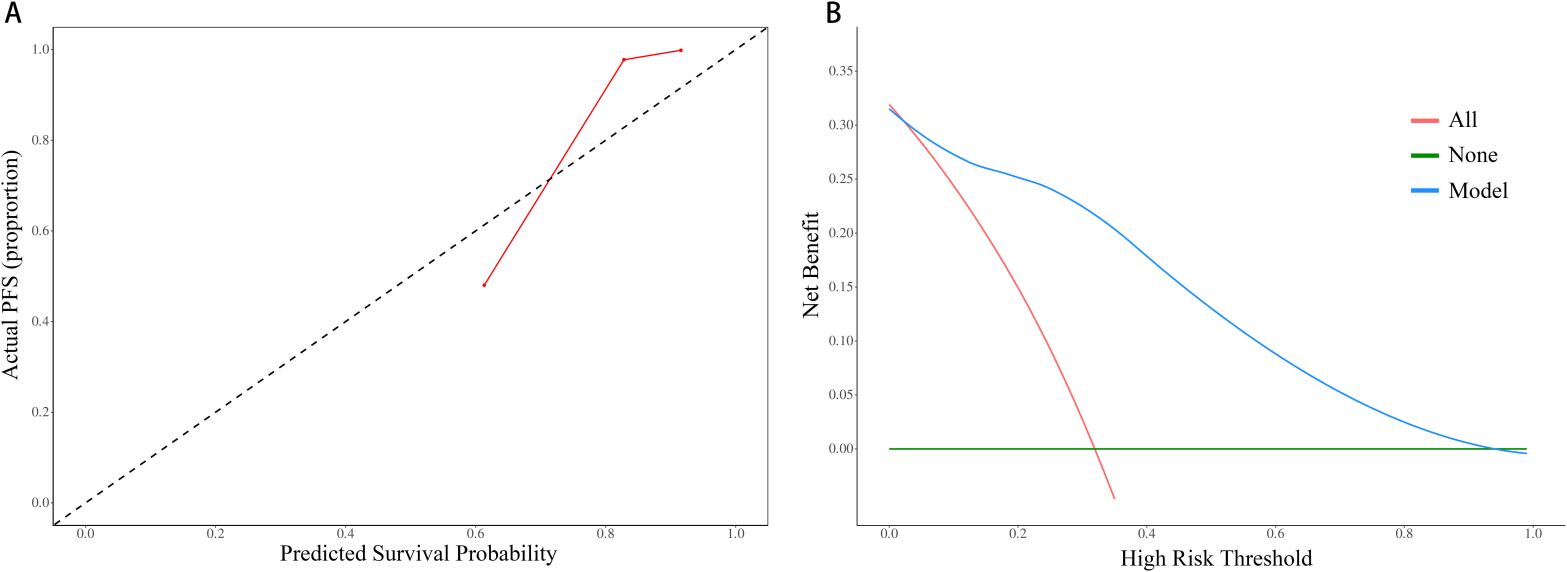
Calibration curve **(A)** and decision curve **(B)** for 18-month progression-free survival.

**Figure 2:**
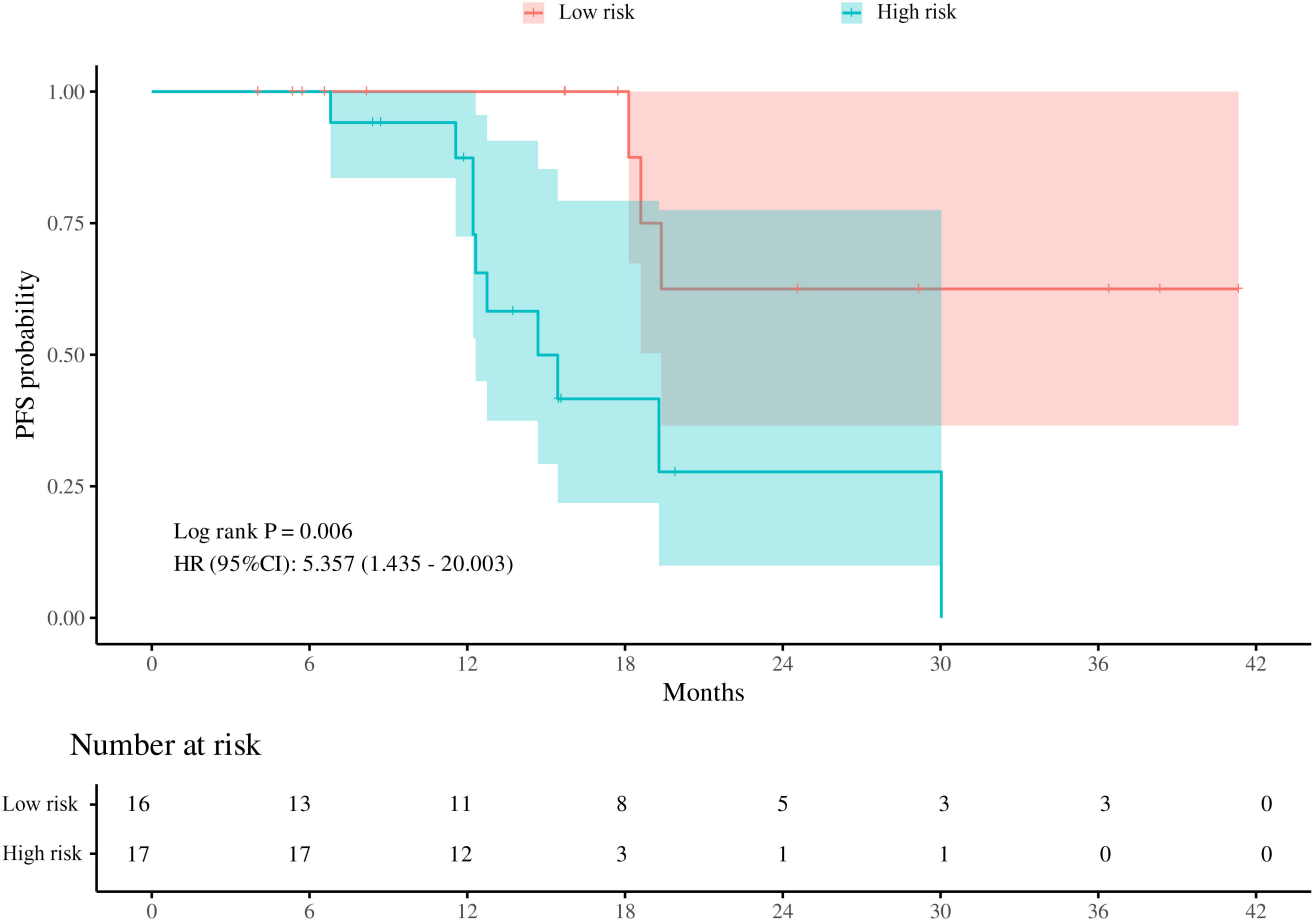
The Kaplan-Meier survival curves for progression-free survival for patients in the high- and low-risk groups.

The C-index of the delta radiomics model for predict progression status at 18 months was 0.78 (95%CI: 0.68-0.88). The calibration curve demonstrated that the delta radiomics model exhibited a high degree of accuracy in predicting PFS at 18 months (**Figure 1A**). The delta radiomics model predicted PFS at 18 months with a high net benefit, as indicated by DCA (**Figure 1B**). The patients were stratified into high- and low-risk categories according to the model’s optimal cutoff value. Kaplan–Meier analysis showed that the high-risk group had significantly shorter PFS than the low-risk group. Cox proportional hazards regression demonstrated that the high-risk group had a significantly higher risk of disease progression than the low-risk group (HR = 5.357, *P* = 0.006) (**Figure 2**).

## Discussion

Recent studies have demonstrated the efficacy of delta radiomics in prognostic studies of various tumors, including pancreatic cancer (25). The predictive value of CT- and MRI-based delta radiomics for the efficacy of NAT treatments in pancreatic cancer has been reported (26, 27). However, the value of this approach in 18F-FDG PET/CT has been documented in a limited number of studies. In this study, we successfully developed and validated a delta radiomics model to estimate PFS in patients with LAPC. The model demonstrated to effectively capture prognostic information from alterations in the dynamics of 18F-FDG PET/CT images, providing significant diagnostic and therapeutic value in the context of LAPC management.

Pancreatic cancer remains a formidable challenge in the field of oncology, with a 5-year survival rate that remains below 5% despite the advent of advanced multimodality therapy regimens, including LAPC (9, 28). The intricacy of LAPC stems from its aggressive biological characteristics, its anatomical proximity to vital vascular structures (e.g., the celiac axis and the superior mesenteric artery), and its limited response to conventional therapeutic modalities (29). Surgical resection is the cornerstone of therapeutic purposes; however, due to vascular involvement, locally advanced pancreatic cancer (LAPC) is often excluded, and NAT is necessary to downstage the tumor and improve resectability (30–32). Current NAT regimens, including FOLFIRINOX and gemcitabine/nab-paclitaxel in combination with radiotherapy, have exhibited encouraging outcomes in converting borderline resectable cases to operable status (33). However, patient prognoses exhibited significant heterogeneity, with median overall and PFS ranges of 10.0 to 32.7 months and 3.0 to 25.3 months, respectively (34). This variability underscores the pressing need for reliable biomarkers that can effectively stratify patients based on their treatment response and prognosis.

Imaging modalities such as contrast-enhanced CT and MRI provide anatomical detail but do not quantify functional tumor activity (35). Therefore, there is an increasing focus on integrating advanced imaging techniques and computational analysis to refine prognostic models. 18F-FDG PET/CT has emerged as a pivotal instrument in the management of pancreatic cancer, attributable to its distinctive capacity to concurrently evaluate metabolic activity and anatomical structure (36). In contrast to conventional imaging modalities, 18F-FDG PET/CT offers a quantitative assessment of glucose metabolism, thereby providing insights into the aggressiveness of tumors and the metabolic changes induced by treatment (37). A multitude of studies have demonstrated a consistent correlation between baseline metabolic parameters, including maximum standardized uptake value (SUVmax) and metabolic tumor volume (MTV), and survival outcomes in pancreatic cancer (38–40). For instance, an analysis by Sara Sheikhbahaei et al (41). demonstrated that patients with elevated peak SUV and high MTV exhibited a 5.45-fold increased risk of mortality (95% CI: 1.76–16.87) compared with patients who presented with both low peak SUV and MTV. Conversely, diminished post-NAT SUVmax levels were associated with a favorable histopathologic response and enhanced resection rates. However, conventional PET/CT metrics are not without their limitations. The metabolic parameters calculated from baseline 18F-FDG PET/CT in this study exhibited unsatisfactory predictive results, indicating their limited value. However, static measurements at a single time point may fail to capture dynamic changes in tumor biology during NAT (25, 42, 43). Additionally, inter-observer variability in manual scoring may compromise the reproducibility of results. These challenges underscore the necessity for automated, quantitative methodologies to enhance predictive precision.

Radiomics, a high-throughput technique for extracting quantitative features from medical images, offers a revolutionary new approach to the diagnosis and treatment of disease by transforming pixel-level data into predictive biomarkers (44). Delta radiomics builds upon this technique by analyzing longitudinal changes in radiological features before and after treatment, thereby capturing temporal heterogeneity in tumor behavior (45, 46). This approach is particularly beneficial in the context of LAPC, where natural anti-programmed cell death (NAT) induced complex microenvironmental changes, such as fibrosis, necrosis, and immune infiltration, that are difficult to observe with conventional imaging modalities. Recent studies have emphasized the superiority of delta radiomics over static models for efficacy prediction in a wide range of diseases (47–49). The present study achieved an accurate prediction of PFS by comparing changes in LAPC on 18F-FDG PET/CT before and after NAT and quantifying these subtle differences using delta radiomics. The Kaplan-Meier curve analysis demonstrated that PFS was significantly shorter in the high-risk group than in the low-risk group, classified according to the optimal thresholds of the delta radiomics model (P = 0.006).

The two features retained in the final model may reflect different aspects of treatment-induced tumor heterogeneity. Wavelet_LHH_firstorder_Kurtosis characterizes the shape of the voxel-intensity distribution after wavelet decomposition. Changes in kurtosis may indicate redistribution of highly and weakly metabolically active voxels following NAT and may therefore be associated with heterogeneous tumor-cell viability, necrosis, fibrosis, or residual metabolically active tumor components. Wavelet_HHH_gldm_DependenceVariance measures the variability of local groups of voxels with similar intensities and may reflect alterations in local texture organization and metabolic complexity. Changes in this feature may be related to treatment-induced stromal remodeling, fibrosis, necrosis, immune infiltration, and the coexistence of treatment-sensitive and treatment-resistant tumor regions. Nevertheless, radiomics features are indirect imaging biomarkers, and their precise biological meaning remains exploratory. Future radiologic-pathologic and molecular correlation studies are required to validate these interpretations.

Inevitably, this study has several limitations. First, the retrospective design and small sample size (n=33) may introduce selection bias and limit generalizability. Although LASSO-Cox regression reduced the dimensionality of the radiomics features and only two delta radiomics features were retained in the final model, the relatively small sample size may still limit the generalizability of our findings. Therefore, external validation in larger multicenter cohorts is urgently needed. Second, external validation of the multicenter cohort is essential to confirm the robustness of the model. Another limitation is that inter-observer and intra-observer reproducibility of tumor segmentation and radiomics feature extraction was not formally assessed using intraclass correlation coefficients. Although all VOIs were delineated by one nuclear medicine physician and reviewed by a senior physician through a consensus-based procedure, the robustness of the selected radiomics features against segmentation variability remains unconfirmed. Future studies should perform repeated segmentation by multiple observers and retain only features demonstrating satisfactory reproducibility. Finally, the results of this study were not applicable to 18F-FDG-negative tumors, as radiomics requires the ability to identify lesions on images. A fixed threshold of 40% of SUVmax was used for tumor segmentation because this approach is straightforward, reproducible, and facilitates the consistent delineation of lesions across pre- and post-treatment PET/CT examinations. Such consistency is particularly important in delta radiomics, in which changes between two imaging time points are quantified. Nevertheless, pancreatic tumors frequently exhibit heterogeneous FDG uptake because of necrosis, fibrosis, inflammation, viable tumor components, and treatment-induced microenvironmental alterations. Therefore, a fixed relative threshold may not fully capture the entire biologically active tumor volume in all patients. Future studies should compare fixed-threshold segmentation with adaptive thresholding, gradient-based methods, and expert manual delineation to determine the optimal segmentation strategy for pancreatic cancer PET radiomics. Future studies should compare different segmentation strategies, such as adaptive thresholding, gradient-based segmentation, or semi-automatic contouring methods, to determine the optimal segmentation approach for PET radiomics analysis in LAPC. Future studies should investigate the predictive value of delta radiomics for LAPC with different immunophenotypes to validate the generalizability of the model.

## Conclusion

18F-FDG PET/CT can be used to dynamically display the response of LAPC to NAT. The delta radiomics model, constructed before and after NAT, can effectively predict PFS, and can be considered a reliable tool with which to better stratify and manage postoperative patients according to their disease risks.

## Data Availability

The data analyzed in this study is subject to the following licenses/restrictions: protect the privacy of patients’ personal information. Requests to access these datasets should be directed to Qiaoliang Chen, https://www.cql9712@163.com.
